# Salt wasting syndrome in brain trauma patients: a pathophysiologic approach using sodium balance and urinary biochemical analysis

**DOI:** 10.1186/s12883-020-01771-8

**Published:** 2020-05-16

**Authors:** Alexandre Lannou, Cedric Carrie, Sebastien Rubin, Gregoire Cane, Vincent Cottenceau, Laurent Petit, Matthieu Biais

**Affiliations:** 1 Anesthesiology and Critical Care Department, CHU Bordeaux, 33000 Bordeaux, France; 2 Univ. Bordeaux Segalen, 33000 Bordeaux, France; 3 Nephrology Department, CHU Bordeaux, 33000 Bordeaux, France

**Keywords:** Salt wasting syndrome, Hyponatremia, Augmented renal clearance, Brain trauma, Intensive care

## Abstract

**Background:**

To explore the underlying mechanisms leading to the occurrence of hyponatremia and enhanced urinary sodium excretion in brain trauma patients using sodium balance and urinary biochemical analysis.

**Methods:**

We conducted a retrospective analysis of a local database prospectively collected in 60 brain trauma patients without chronic renal dysfunction. Metabolic and hemodynamic parameters were averaged over three consecutive periods over the first seven days after admission. The main outcome investigated in this study was the occurrence of at least one episode of hyponatremia.

**Results:**

Over the study period, there was a prompt decrease in sodium balance (163 ± 193 vs. -12 ± 154 mmol/day, *p < 0.0001*) and free water clearance (− 0.7 ± 0.7 vs. -1.8 ± 2.3 ml/min, *p < 0.0001*). The area under the ROC curves for sodium balance in predicting the occurrence of hyponatremia during the next period was 0.81 [95% CI: 0.64–0.97]. Variables associated with averaged urinary sodium excretion were sodium intake (R^2^ = 0.26, *p < 0.0001*) and fractional excretion of urate (R^2^ = 0.15, *p = 0.009*). Urinary sodium excretion was also higher in patients with sustained augmented renal clearance over the study period (318 ± 106 vs. 255 ± 135 mmol/day, *p = 0.034*).

**Conclusion:**

The decreased vascular volume resulting from a negative sodium balance is a major precipitating factor of hyponatremia in brain trauma patients. Predisposing factors for enhanced urinary sodium excretion were high sodium intake, high fractional excretion of urate and augmented renal clearance over the first seven days after ICU admission.

## Background

Salt wasting syndrome (SWS) is a common cause of hyponatremia in the brain trauma patients, with a reported incidence varying from 0.8 to 34.6% according to the definition [1]. The most widespread theory explaining the renal loss of sodium purports a neurohumoral response involving a pressure-induced natriuresis, an increased level of natriuretic peptides and a hypoaldosteronism syndrome [2, 3]. Some authors also suggested a defect in proximal tubular sodium reabsorption, as suggested by the increased excretion fraction of solutes exclusively transported in the proximal tubule [4].

Although the pathophysiologic mechanisms responsible for SWS remain mainly unknown, the decreased vascular volume resulting from a renal loss of sodium is thought to be the leading mechanism for an enhanced ADH secretion despite an increasing plasma hypoosmolality [2, 4]. Determining volume status is thus of paramount importance in brain-trauma patients, the assessment of which being universally agreed to be inaccurate by usual clinical criteria [3].

The hypothesis pertaining to this study is that a negative sodium balance could be a precipitating factor of hyponatremia during the acute phase of traumatic brain injury. Our objective was thus to explore the underlying mechanisms leading to the occurrence of hyponatremia and enhanced urinary sodium excretion in brain trauma patients using sodium balance and urinary biochemical analysis.

## Methods

### Design, population and settings

This study is a retrospective analysis of our local database (declared to the French Data Protection Authority, number 2166637v0) prospectively collected over an 8-month period (June 2018 to January 2019) in every brain trauma patient (Abbreviated Injury Score [AIS] > 3) consecutively admitted in a 25-bed Surgical and Trauma Intensive Care Unit (ICU). Study participants had to have an ICU length of stay alive ≥5 days and no evidence of congestive heart failure, cirrhosis or chronic renal failure (measured creatinine clearance [CL_CR_] < 60 ml/min/1.73m^2^). Patients who presented hyponatremia (i.e. ≤ 135 mmol/L) at ICU admission were also excluded.

As previously described, all patients were managed by a standardized protocol in accordance with French recommendations [5, 6]. Transient osmotherapy was reserved for refractory intracranial hypertension with signs of brain herniation (100 mL of 20% Mannitol bolus if Natremia > 150 mmol/L; 100 ml of Hypertonic saline 10% given over 1 h otherwise). Hyperventilation was prohibited. Patients who presented at least one episode of hyponatremia underwent Brain Natriuretic Peptides (BNP, normal range < 100 pg/ml), aldosterone (normal range 53–645 pmol/L), Thyroid-Stimulating Hormone (TSH, normal range 0.35–4.94 UI/L) and cortisol (normal range 100–540 nmol/L) levels measurements and were treated by administration of hypertonic saline (NaCL 10% administered continuously, 1 g/hour until normalization of Natremia and then adapted to the sodium balance). Fludrocortisone (100–400 μg/day) was used in refractory cases of SWS [7].

According to the French Data Protection Authority, the handling of these data for research purposes was declared to the Data Protection Officer of the University Hospital of Bordeaux. The observational character of the present study was confirmed from Ethics Committee of the French Society of Anesthesiology and Intensive Care (IRB number: CERAR 00010254–2018-153), which waived the need for written or oral consent. The patients and/or next of kin were informed about the potential inclusion of their anonymized data for retrospective studies, and none expressed opposition.

### Study protocol and data collection

Plasma and 24-h urinary samples were recorded daily and CL_CR_ was calculated as follows: $\frac{24\ \mathrm{hr}\ \mathrm{urinary}\ \mathrm{volume}\ \mathrm{x}\ \mathrm{urinary}\ \mathrm{creatinine}}{\mathrm{plasma}\ \mathrm{creatinine}\ }$, converted in ml/min and normalized to a body surface area of 1.73 m^2^ (Dubois formula). Sustained augmented renal clearance was considered in patients who presented a mean CL_CR_ > 130 ml/min/1.73m^2^ over the study period [6]. Free water clearance (CL_H20_) was also calculated as follows: 24-h urine volume x (1 - $\frac{\mathrm{Urinary}\ \mathrm{osmolality}\ \left(\mathrm{mmol}/\mathrm{kg}\right)}{\mathrm{Plasma}\ \mathrm{osmolality}\ \left(\mathrm{mmol}/\mathrm{kg}\right)}$), converted in ml/min.

Fractional excretion of sodium (FE_Na_, normal range < 1%) and urate (FE_urate_, normal ranges < 11%) were both calculated with standard formulas. Fluid balance, sodium balance and averaged hemodynamic data (mean arterial pressure [MAP], norepinephrine [NE] infusion) were collected during the first seven days after admission. Metabolic and hemodynamic parameters were averaged over three consecutive periods: an early (day 1 – day 2), intermediate (day 3–4) and a late period (day 5 to day 7). The daily sodium intake conversion table is reported in Supplementary Data. The 7-day period was arbitrarily defined according to the risk of intracranial hypertension justifying close monitoring, prevention and rapid correction of hyponatremia.

### Statistical analysis

Results are expressed as mean ± standard deviation or median (25 to 75% interquartile range) for continuous variables and as numbers (percentages) for categorical variables. The data distribution was analyzed by a Kolmogorov-Smirnov test.

The main outcome investigated in this study was the occurrence of at least one episode of hyponatremia during the first seven days after admission for TBI. Only the first episode of hyponatremia was considered over the first seven days after admission. As the occurrence of hyponatremia justified a prompt increase in sodium intake, sodium balances couldn’t be compared over the same period between patients who presented or not an episode of hyponatremia. Hemodynamic and metabolic parameters of patients who presented or not a first episode of hyponatremia during the intermediate and late periods were thus compared during the previous period. The association between sodium balance during one period and the occurrence of hyponatremia the following period was also assessed using a receiving operator curve (ROC) analysis. A sample size of 56 patients was necessary to assess the predictive value of sodium balance with an AUC ≥ 0.75 and different from 0.5, assuming a 5% type I error rate, an 80% power and a prevalence of hyponatremia ≥25% [8]. A threshold analysis was also performed using a grey zone approach [9].

Precipitating factors of hyponatremia were also described by comparison of metabolic and hemodynamic parameters during the 48 h before the occurrence of hyponatremia. Continuous variables were compared using Wilcoxon test for paired samples and categorical variables were compared using the chi-square test or Fisher’s exact test as appropriate. For the secondary outcome of this study, linear regression models were used to assess the effect of sodium intake, CL_CR_, MAP and FE_urate_ on urinary sodium excretion averaged over the study period. A *p* value < 0.05 was considered statistically significant.

Statistical analyses were performed using XLSTAT 2017 for Windows (Addinsoft Paris, France).

## Results

### Characteristics of the population

Over the study period, 60 TBI patients without chronic renal dysfunction contributed to the database. Overall, 16 patients (27%) presented at least one episode of hyponatremia, occurring after a median duration of 6 [4–7] days after ICU admission. Each episode of hyponatremia was adequately treated by administration of hypertonic saline ± fludrocortisone. The characteristics of the population are resumed Table 1.

**Table 1:** Characteristics of the population

	Overall population (***N*** = 60)	Control group (***N*** = 44)	Hyponatremia day 1–7 (***N*** = 16)	***p***
**Demographic data**				
• Age (years)	48 [32–60]	48 [32–61]	49 [22–59]	*0.627*
• Male sex	53 (88)	39 (89)	14 (88)	*0.903*
• Weight (kg)	76 [67–88]	78 [69–90]	72 [60–83]	*0.143*
**Comorbidities**				
• Chronic hypertension	12 (20)	9 (20)	3 (19)	*0.957*
• Diabetes mellitus	3 (5)	3 (7)	0 (0)	*0.501*
• Previous use of ACE inhibitors	5 (8)	3 (7)	2 (13)	*0.876*
• Previous use of β-blockers	3 (5)	2 (3)	1 (6)	*0.927*
**Reason for admission**				
• Isolated TBI • Multiple trauma with TBI	26 (43) 34 (57)	17 (39) 27 (61)	9 (56) 7 (44)	*0.223* *0.223*
**Prognostic scores**				
• Initial GCS	9 [5–14]	10 [6–14]	7 [4–12]	*0.213*
• SAPS II	45 [36–52]	44 [36–50]	39 [45–53]	*0.484*
**Neurosurgical procedures**				
• Intracranial pressure monitoring	35 (58)	27 (57)	10 (63)	*0.693*
• External CSF derivation	16 (27)	10 (23)	6 (38)	*0.253*
• Parietal craniotomy	17 (28)	14 (32)	3 (19)	*0.321*
• Craniectomy	9 (15)	7 (16)	2 (13)	*0.744*
**Intracranial hypertension**	24 (40)	14 (33)	9 (56)	*0.097*
**Management in the ICU**				
• Use of mechanical ventilation	58 (97)	41 (95)	16 (100)	*0.380*
• Use of vasopressors	56 (93)	40 (93)	15 (94)	*0.921*
• Use of osmotherapy	17 (28)	10 (23)	7 (44)	*0.122*
• Use of hypothermia	17 (28)	10 (23)	6 (38)	*0.274*
• Use of barbiturates	14 (23)	8 (19)	5 (31)	*0.297*
• Use of NSAIDs	10 (17)	8 (19)	2 (13)	*0.578*
• Use of hydrocortisone	6 (10)	6 (14)	0 (0)	*0.115*
• Use of desmopressine	4 (7)	3 (7)	1 (6)	*0.921*
**Mean fluid and sodium balances**				
• Mean fluid balance (L)	0.8 [0.6–1.2]	0.9 [0.5–1.2]	0.6 [0.6–0.9]	*0.115*
• Mean sodium balance (mmol)	41 [− 33–104]	41 [−7–104]	29 [−41–100]	*0.621*
• Loss of weight	0 [−1–2]	0 [−2 − + 2]	0 [− 1–0]	*0.843*
**Outcome**				
• Death during the ICU stay	7 (12)	3 (7)	4 (25)	*0.052*
• Duration under ventilation	9 [5–17]	9 [3–17]	10 [7–15]	*0.412*
• ICU length of stay	16 [11–28]	15 [11–26]	18 [13–24]	*0.616*

Results expressed as number (percentage) or median [interquartile 25–75]

Averaged metabolic and hemodynamic parameters over the study periods are described Table 2. Over the study period, there was a prompt decrease of sodium balance (163 ± 193 vs. -12 ± 154 mmol/day, *p < 0.0001*), induced by an enhanced sodium excretion (202 ± 183 vs. 316 ± 154 mmol/day, *p = 0.000*2) and a decrease in sodium intake (375 ± 110 vs. 304 ± 114 mmol/day, *p = 0.0003*). Similarly, the decreased fluid balance (1.3 ± 1.0 vs. 0.6 ± 0.9 L/day, *p = 0.001*) was induced by an enhanced diuresis (2.5 ± 0.8 vs. 2.0 ± 1.1 L/day, *p = 0.003*) and a decreased fluid intake (3.3 ± 0.8 vs.3.1 ± 0.9 L/day, *p = 0.038*). Finally, a decreased plasma osmolality (307 ± 12 vs. 303 ± 12 mOsm/L, *p = 0.014*) and increased urinary osmolality (523 ± 141 vs. 605 ± 124 mOsm/L, *p = 0.003*) contributed for a decreased free water clearance over time (− 0.7 ± 0.7 vs. -1.8 ± 2.3 ml/min, *p < 0.0001*).

**Table 2:** Averaged hemodynamic and metabolic parameters during the study period in the overall population

	Study period	Day 1–2	Day 3–4	Day 5–7
**Natremia (mmol/L)**	142 [139–144]	142 [140–146]	143 [140–147]	140 [138–143] *^,^*†*
**Hyponatremia (Na ≤ 135 mmol/)**	16 (27)	0 (0)	5 (8)	11 (18) *
**Fluid intake (L/24 h)**	3.2 [2.8–3.6]	3.3 [2.9–3.8]	3.2 [2.7–3.7]	3.2 [2.5–3.7] *
**Diuresis (L/24 h)**	2.2 [1.9–2.8]	1.8 [1.3–2.5]	2.2 [1.6–2.9]	2.4 [1.8–2.9] *
**Fluid balance (L/24 h)**	0.8 [0.6–1.2]	1.4 [0.4–2.0]	1.0 [0.4–1.4]	0.6 [− 0.1–1.1] *
**Sodium intake (mmol/24 h)**	323 [277–378]	363 [299–432]	295 [234–371]	302 [229–365] *
**Urinary sodium excretion (mmol/24 h)**	284 [221–362]	132 [91–275]	309 [187–422]	310 [226–409] *
**Sodium balance (mmol/24 h)**	47 [− 33–104]	215 [84–293]	16 [− 115–106]	22 [− 85–90] *
**24-h CL**_**CR**_**(ml/min/1.73m**^**2**^**)**	143 [116–160]	136 [96–154]	143 [110–165]	147 [115–166] *
**Augmented renal clearance**	32 (53)	33 (55)	34 (57)	33 (55)
**Urinary osmolality (mmol/L)**	569 [503–653]	498 [423–627]	590 [487–655]	603 [510–700] *
**24-h CL**_**H2O**_**(ml/min/1.73m**^**2**^**)**	−1.3 [− 1.5 – − 1.0]	− 0.8 [− 1.2 – − 0.4]	− 1.3 [− 1.6 – − 0.8]	− 1.6 [− 2.1 – − 1.1] *^,^*†*
**Fractional excretion of sodium (%)**	0.94 [0.76–1.21]	0.56 [0.33–1.15]	0.98 [0.70–1.40]	0.94 [0.72–1.31] *
**Fractional excretion of urate (%)**	19 [12–22]	13 [11–18]	16 [12–21]	22 [13–28] *^,^*†*
**Mean MAP**	92 [87–96]	87 [83–92]	91 [85–97]	93 [88–100] *
**Use of vasopressor**	54 (90)	54 (90)	42 (70)	30 (50) *

Data expressed as median [interquartile 25–75] and numbers (percentages)

* = *p* < 0.05 between period 1 and period 3; † = *p* < 0.05 between period 2 and period 3 (continuous variables compared using Wilcoxon test for paired samples)

### Precipitating factors for hyponatremia in brain trauma patients

The evolution of metabolic and hemodynamic parameters during the 48 h before the occurrence of hyponatremia are depicted Table 3. The rapid fall in Natremia was associated with an increased natriuresis, a negative sodium balance and a significant hemoconcentration over the two previous days. The day of hyponatremia, each patient presented normal values of BNP (41 [26–80] pg/ml), aldosterone (90 [72–180] pmol/L), TSH (0.6 [0.5–1.4] UI/L) and cortisol (396 [308–505] nmol/L).

**Table 3:** Evolution of hemodynamic and metabolic parameters in patients who presented a first episode of hyponatremia over the study period (*N* = 16)

	Day of hyponatremia	Day - 1	Day - 2
**Natremia (mmol/L)**	135 [134–135]	138 [137–139]	141 [139–144] *^,^*†*
**Fluid intake (L/24 h)**	3.0 [2.5–3.3]	2.5 [2.3–3.0]	2.7 [2.6–3.0]
**Diuresis (L/24 h)**	2.2 [2.0–3.1]	2.3 [1.7–3.2]	2.2 [1.9–2.6]
**Fluid balance (L/24 h)**	0.6 [− 0.1–1.0]	0.5 [− 0.8–0.9]	0.4 [−0.2–1.0]
**Sodium intake (mmol/24 h)**	356 [297–425]	278 [224–363]	288 [252–319] *
**Urinary sodium excretion (mmol/24 h)**	316 [178–452]	372 [288–553]	403 [297–466]
**Sodium balance (mmol/24 h)**	87 [− 97–148]	− 98 [− 211–74]	−130 [− 213 – − 2]
**24-h CL**_**CR**_**(ml/min/1.73m**^**2**^**)**	145 [127–165]	161 [139–173]	141 [128–163]
**Urinary osmolality (mmol/L)**	595 [491–686]	551 [498–712]	636 [483–740]
**24-h CL**_**H2O**_**(ml/min/1.73m**^**2**^**)**	- 1.6 [−1.9 – − 1.3]	- 1.6 [− 1.9 – − 1.3]	- 1.8 [− 2.0 – − 1.1]
**Fractional excretion of sodium (%)**	1.04 [0.52–1.80]	0.95 [0.81–2.27]	1.15 [0.99–1.63]
**Fractional excretion of urate (%)**	27 [15–28]	21 [14–27]	23 [16–25] *
**BUN (mmol/L)**	4.9 [3.3–6.3]	4.1 [2.5–5.2]	3.0 [2.3–4.6] *
**Protidemia (g/L)**	65 [60–67]	62 [58–68]	58 [53–63] *^,^*†*
**Hte (%)**	32 [29–36]	33 [30–35]	31 [30–35] *†*
**Mean MAP (mmHg)**	92 [86–98]	94 [91–94]	91 [87–94]
**Use of vasopressor**	5 (31)	8 (50)	8 (50)

Data expressed as median [interquartile 25–75] and numbers (percentages)

* = *p* < 0.05 between Day − 2 and Day 0; † = *p* < 0.05 between Day − 2 and Day − 1 (continuous variables compared using Wilcoxon test for paired samples)

**Fig. 1:**
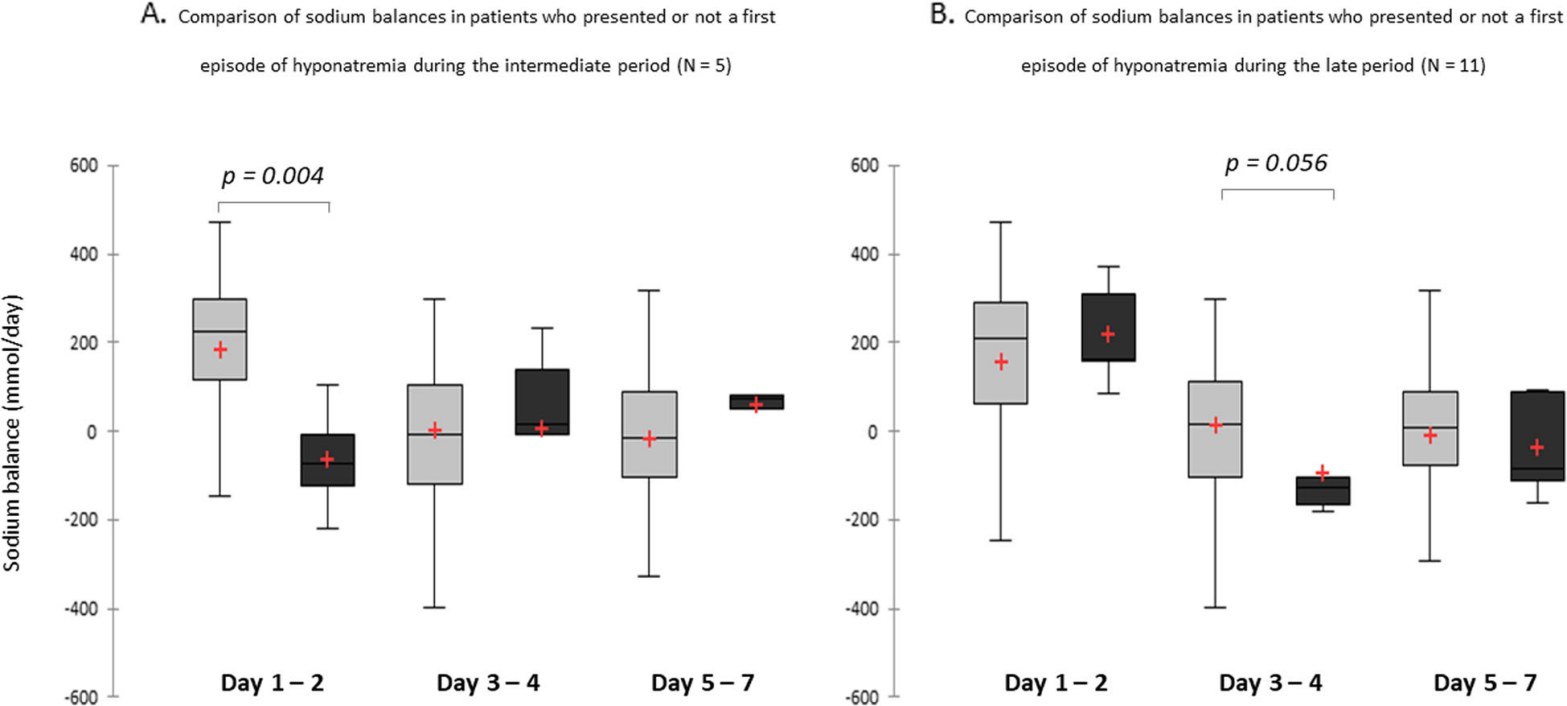
Comparison of sodium balances in patients who presented or not a first episode of hyponatremia (**a**) during the intermediate period [day 3–4] or (**b**) during the late period [day 5–7]. *Dark grey: patients with hyponatremia; light grey: control group*

Patients who presented an episode of hyponatremia during the intermediate and late periods had lower sodium balances during the previous period (Fig. 1). There was no statistical difference regarding other averaged metabolic or hemodynamic data.

**Fig. 2:**
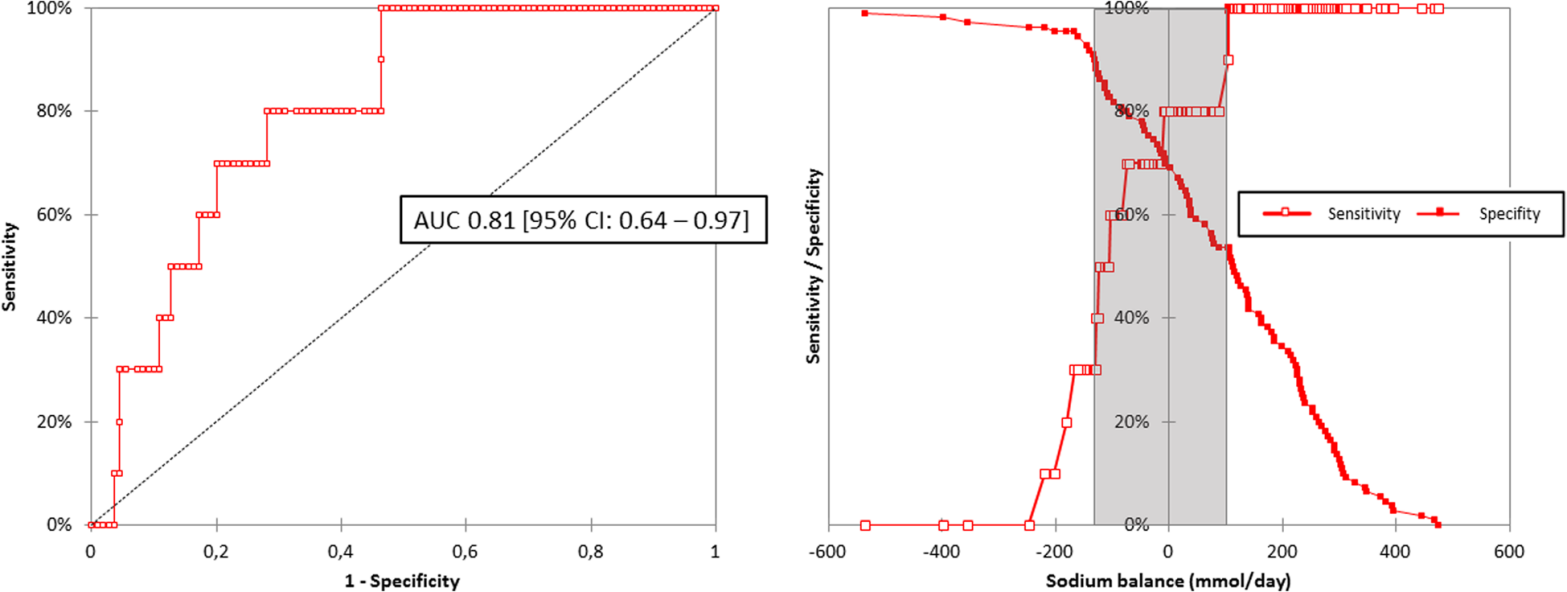
Ability of sodium balance during one period to predict the occurrence of hyponatremia the following period: ROC curve andTwo-curve graph showing the sensitivity and specificity of the different values of sodium balance to predict the occurrence of hyponatremia the following period; the inconclusive grey zone is displayed as a grey rectangle for a sodium balance between − 130 and + 100 mmol/day

A negative sodium balance during one period was associated with an increased risk of hyponatremia the next period (OR = 9.3 [2.2–40.5], *p = 0.001*). A negative sodium balance had a sensibility of 80% [95%CI: 48–95%] and a specificity of 69% [95%CI: 59–77%] to predict hyponatremia the next period. The area under the ROC curves for sodium balance over one period in predicting the occurrence of hyponatremia during the following period was 0.81 [95% CI: 0.64–0.97] (Fig. 2). The rate of patients who belonged to the grey zone was 38%.

### Predisposing factors for enhanced sodium excretion

The averaged urinary sodium excretion was high, but not statistically different in patients who presented or not one episode of hyponatremia over the study period (287 ± 90 vs. 282 ± 133 mmol/day, *p = 0.621*). Continuous variables associated with averaged urinary sodium excretion were sodium intake (R^2^ = 0.26, *p < 0.0001*) and FE_urate_ (R^2^ = 0.15, *p = 0.009*). Urinary sodium excretion was also higher in patients with sustained augmented renal clearance over the study period (318 ± 106 vs. 255 ± 135 mmol/day, *p = 0.034*). The mean MAP was high, but not statistically associated with renal sodium excretion (R^2^ = 0.035, *p = 0.151*).

## Discussion

To our knowledge, this is the first study aiming to assess the predisposing and precipitating factors of hyponatremia in brain trauma patients. Over the first seven days after ICU admission, there was an inverse relationship between the prompt increase in urinary sodium excretion and the decrease in free-water clearance. Predisposing factors for enhanced urinary sodium excretion were higher sodium intake, higher FE_urate_ values and augmented renal clearance over the first seven days after ICU admission. Patients with negative sodium balance and hemoconcentration were particularly at risk for hyponatremia during this period.

On the one hand, there is general agreement that the renal loss of sodium is responsible for variable reduction in extracellular volume (ECV), leading to an appropriate ADH secretion that may promote renal free-water retention and hyponatremia despite an increasing plasma hypoosmolality [2, 4]. In this context, Audibert et al. determined blood volume by gold-standard radioisotope dilution methods and demonstrated a significant reduction in ECV after subarachnoid hemorrhage [3]. Authors argued that hyponatremia might be prevented by controlled sodium intake adapted to renal excretion. It should be noticed that the complexity of sodium intake calculation may preclude any clinical application. In this context, our results may suggest that monitoring of hemoconcentration markers (such as BUN or protidemia) should promote a prompt increase in ECV and sodium intake to avoid a sustained plasma hypoosmolality.

On the other hand, the pathophysiology of the enhanced urinary sodium excretion is not completely understood. Some authors previously advocated a decreased sympathetic outflow and an increased level of circulating natriuretic peptides [10, 11]. Moreover, hypoaldosteronism has been considered as a contributing factor for the enhanced sodium excretion [3]. Our results didn’t corroborate this neurohumoral theory, with normal BNP and aldosterone values reported in patients experiencing a first episode of hyponatremia. This result was in accordance with Maesaka et al. who first suggested that the contribution of natriuretic factors or renin-aldosterone levels was not straightforward in brain trauma patients [4].

According to our results, an enhanced tubular fluid output associated with a proximal sodium reabsorption defect should be considered as the leading mechanisms explaining the marked increase in urinary sodium excretion.

- After the initial resuscitation, the high sodium intake and increased sympathetic stimulation may be responsible for a delayed pressure-induced natriuresis [12]. Our results are not inconsistent with this hypothesis since higher MAP were observed over time despite a progressive norepinephrine withdrawal, probably explained by cessation of anesthetic agents and/or weaning from mechanical ventilation. The hemodynamic compensatory responses allow reaching a new steady state sodium balance at a lower extracellular volume, depending on the extend of renal loss and sodium input [10].
- Moreover, perturbations in renal hemodynamics may lead to an enhanced glomerular filtration rate which may be considered as a contributing factor for the enhanced tubular fluid output [13]. Our results are in accordance with a previous report suggesting a significant relationship between 24-CL_CR_ and urinary osmole excretion over the first days after TBI [14]. The hypothesis underlying these results was that the marked increase in GFR should be associated with an increased tubular fluid output, responsible for an enhanced diuresis and osmole excretion.
- Finally, the association between high FE_urate_ and enhanced sodium excretion over time may comfort previous studies that hypothesized a defect in sodium transport in the proximal tubule [4]. In this respect, Maesaka et al. first suggested the increased excretion fraction of solutes exclusively transported in the proximal tubule such as lithium, urate and eventually phosphate in patients with SWS [15–17]. Moreover, proximal renal tubular dysfunction has recently been described in brain-damaged patients [18]. This study thus reinforces the multifactorial pathophysiology of SWS which deserves further investigations.

Our study was impaired by several limitations. First, we acknowledge a limited number of patients retrospectively included, although contributing for numerous data collected over the first seven days after admission. This period was arbitrarily chosen and thus may under-estimate the incidence of hyponatremia in brain trauma patients. Moreover, hormonal status could not be compared between patients with or without hyponatremia. Although patients with hyponatremia displayed normal values of BNP and aldosterone, the neurohumoral theory can’t be formally refuted. Finally, we didn’t aim to differentiate SWS and SIADH as those two entities share several serum and urinary biological criteria. In this context, we acknowledge the lack of specific assessment of the extracellular volume. On the other hand, no patient required water restriction to correct hyponatremia, which is not suggestive for SIADH in this context.

## Conclusion

The decreased vascular volume resulting from a negative sodium balance should be considered as a precipitating factor of hyponatremia in brain trauma patients. Predisposing factors for enhanced urinary sodium excretion were mean sodium intake, mean FE_urate_ values and augmented renal clearance over the first seven days after ICU admission. Further studies are needed to corroborate the hypothesis pertaining to this study.

## Supplementary information

**Addtional file 1**. Daily sodium intake conversion table.

## Data Availability

The datasets used and/or analyzed during the current study are available from the corresponding author on reasonable request.

## Abbreviations

ADH: Anti-Diuretic Hormone
AIS: Abbreviated Injury Score
AUC: Area Under Curve
BNP: Brain Natriuretic Peptides
BUN: Blood Urea Nitrogen
CL_CR_: Creatinine Clearance
CL_H2O_: Free Water Clearance
ECV: Extracellular Volume
FE_Na_ / FE_urate_: Fractional Excretion of Sodium / Urate
ICU: Intensive Care Unit
MAP: Mean Arterial Pressure
NE: Norepinephrine
ROC: Receiving Operator Curve
SWS: Salt wasting syndrome
TSH: Thyroid-Stimulating Hormone
