# Supplementary material for: Salt wasting syndrome in brain trauma patients: a pathophysiologic approach using sodium balance and urinary biochemical analysis
Source: BMC Neurol. 2020 May 16;20:190. doi: 10.1186/s12883-020-01771-8 (PMC7229604; doi:10.1186/s12883-020-01771-8)
Supplement: Supplementary file 1 — Addtional file 1. Daily sodium intake conversion table. [file 12883_2020_1771_MOESM1_ESM.pdf]

Supplementary Data 1. Daily sodium intake conversion table

Unit per day	Na (mmol/unit)
**Fluid intakes**	
Non-balanced Crystalloid Solutions	
- *NaCL 0,9% (per ml)*	*0.15*
- *NaCL 10% (per ml)*	*1.71*
- *NaCL 3%*	*0.51*
- *PG5 (per ml)*	*0.07*
Balanced Crystalloid Solutions	
- *Isofundine (per ml)*	*0.15*
- *Ringer lactate (per ml)*	*0.13*
Bicarbonates	
- *Bicarbonates 1,4% (per ml)*	*0.17*
- *Bicarbonates 4,2% (per ml)*	*0.50*
Albumin (4 or 20%, per ml)	*0.15*
Parenteral Nutrition (per ml)	*0.04*
Enteral Nutrition (per ml)	*0.04*
**Antibiotic agents**	
- *Amoxicillin ± Clavulanate (per 2g unit)*	*5.48*
- *Cefazolin (per 2g unit)*	*4.40*
- *Cefotaxime (per 2g unit)*	*4.20*
- *Ceftriaxone (per 2g unit)*	*7.20*
- *Ceftazidime (per 2g unit)*	*4.50*
- *Ciprofloxacin (per 200mg/100ml unit)*	*30.80*
- *Levofloxacin (per 500mg/100ml unit)*	*15.80*
- *Linezolid (per 600mg/300ml unit)*	*4.95*
- *Meropenem (per 2g unit)*	*7.80*
- *Metronidazole (per 500mg/100ml unit)*	*14.76*
- *Piperacillin ± Tazobactam(per 4g unit)*	*11.35*
**Other treatments**	
- *Levetiracetam (per 500 mg unit)*	*0.80*
- *Paracetamol (per 1g/100ml unit)*	*0.17*
- *Phosphoneuros (per 100 drops)*	*15.52*
- *Thiopental (per 1g unit)*	*4.60*
